# Risk of establishment of canine leishmaniasis infection through the import of dogs into South Africa

**DOI:** 10.4102/ojvr.v86i1.1634

**Published:** 2019-05-28

**Authors:** Abdalla A. Latif, Bonginkosi Nkabinde, Brian Peba, Olivier Matthee, Ronel Pienaar, Antoinette Josemans, Daniel Marumo, Karien Labuschagne, Nada A. Abdelatif, Andreas Krüger, Ben J. Mans

**Affiliations:** 1 School of Life Sciences, University of KwaZulu-Natal, Durban, South Africa; 2 Epidemiology, Parasites and Vectors, Agricultural Research Council-Onderstepoort Veterinary Research, Pretoria, South Africa; 3 Medical Research Council, Durban, South Africa; 4 Department of Tropical Medicine, Bundeswehr Hospital, Hamburg, Germany; 5 Department of Life and Consumer Sciences, College of Agriculture and Environmental Sciences, University of South Africa, Johannesburg, South Africa; 6 Department of Tropical Veterinary Diseases, Faculty of Veterinary Sciences, University of Pretoria, Pretoria, South Africa

**Keywords:** canine leishmaniasis, risk assessment, Phlebotominae, ticks, South Africa

## Abstract

Canine leishmaniasis is a vector-borne disease caused by protozoa of the genus *Leishmania* that affect dogs, humans and wildlife. Sandflies of the genera *Phlebotomus* and *Lutzomyia* are the primary vectors. Canine leishmaniasis is an exotic and controlled disease in South Africa. The main purpose of our risk assessment study was to evaluate the likelihood that this exotic disease could enter and be established in South Africa through importation of live dogs. Risk analysis to the spread of the disease follows the World Organization for Animal Health (OIE) formal method of quantitative risk assessment documented as a step-by-step process. We have identified and discussed 11 possible risk factors involved in three steps for final assessment. The annual average number of diagnostic tests performed on imported dogs from 44 countries for 2011–2015 was 1158. Leishmania is reported to occur in 21/44 (47.7%) exporting countries. A total of 71.1% of *Leishmania* positive dogs were imported from these endemic countries. The yearly percentage of *Leishmania* positive dogs ranged from 0.2% to 2%. Three confirmed clinical and fatal cases of leishmaniasis in dogs of unidentified origin have been reported by our laboratory and the state veterinarians. The disease has been reported in neighbouring countries as well as the putative sandfly vectors. This study concluded that the risk for the introduction and degree of uncertainty of *Leishmania* in imported dogs in South Africa are moderate. Risk mitigation and recommendations such as investigations into possible occurrence of autochthonous leishmaniasis in the country, surveillance in its wildlife reservoirs and systematic surveillance of sandfly populations are discussed.

## Introduction

Canine leishmaniasis (CanL) is a vector-borne disease caused by protozoa of the genus *Leishmania* (Kinetoplastida: Trypanosomatidae) that affect dogs in tropical and subtropical regions of the Old World (Africa, Asia, and Southern Europe) and the New World (South America and North America) (Dantas-Torres 2009; Otranto & Dantas-Torres 2009). The most important species of *Leishmania* for dogs is *Leishmania infantum* (syn. *Leishmania chagasi*), which causes visceral and cutaneous diseases in humans (Baneth et al. 2008; Duprey et al. 2006). Can L is expanding its geographical range from northern Argentina to the northern United States covering 18 states and southern Canada (Duprey et al. 2006). In Europe, the extension of the range has been reported in Italy, where there is evidence that CanL is currently expanding into continental climate areas of northwestern Italy, far from the recognised disease-endemic areas along the Mediterranean coasts (Ferroglio et al. 2005). Leishmaniasis is one of the major infectious diseases afflicting the world’s poorest populations living mainly in rural and suburban areas (Alvar, Yactayo & Bern 2006). Sandflies of the genera *Phlebotomus* are the vectors in the Old World and *Lutzomyia* in the New World, that is, insects responsible for parasite transmission. Progress in phlebotomine sandfly research that included taxonomy and systematics, vector competence, eco-epidemiology and vector control has recently been reviewed (Bates et al. 2015). The prevalence of canine *Leishmania* infection in endemic areas is considerably higher than that of apparent clinical illness (Baneth et al. 2008), indicating that not all infected dogs develop the disease. This has also been demonstrated by an experimental model of infection, which showed that dogs developed variable immune responses, and although some became sick, other infected dogs remained clinically healthy over 5 years of observation (Pinelli et al. 1994).

The immunofluorescence antibody test (IFAT), which uses whole promastigote antigen, is highly specific and sensitive for the detection of exposure to *Leishmania*, but may lack sensitivity to detect infected but clinically healthy dogs (Mettler et al. 2005). Although 88% – 100% of the dogs showing clinical signs were found serologically positive, only 30% – 66% of the subclinically infected ones were positive (Porrozzi et al. 2007; Solano-Gallego et al. 2001). Dogs with symptomatic as well as asymptomatic infections are infectious to sandflies. However, infectiousness was shown to be higher in dogs with clinical disease (Baneth et al. 2008). Infected dogs in non-endemic areas may also contribute to the maintenance of the *Leishmania* parasites within the canine population through possible non-sandfly vector transmission modes of infection as documented in hematophagous ectoparasites, such as ticks (Solano-Gallego et al. 2009). Recently, three species of the sandfly genus *Sergentomyia* were demonstrated to have a high rate of *L. infantum*-positive females under natural conditions. This finding indicated that these species were the natural vectors of CanL in the Mont-Rolland area of Senegal and contradicts the notion that species of the *Phlebotomus* genus in the Old World are the only vectors of leishmaniasis (Senghor et al. 2016). *Leishmania* vertical infection was demonstrated experimentally in puppies born to infected female and male beagles, and transmission was assumed to be transplacental (Boggiatto et al. 2011; Rosypal et al. 2005). Transplacental infection of litters in dog breeding may be the most important mechanism of transmission (Duprey et al. 2006). The occurrence of *L. infantum* infection in dogs in 18 states in the United States and two provinces in Canada where the vector sandflies have not been identified is now well established (Duprey et al. 2006).

Research into the sandfly populations of South Africa has never been carried out systematically and the species records came out from only ad hoc and limited surveys. There are about 17 species of Phlebotomines known in South Africa and the last record dates back to 1987 (Braack et al. 1981; Davidson 1979, 1980, 1987; Lewis 1967, 1971; Zielke 1971); however, not much is known about their distribution and their ecology, biology and disease relationships. A case of cutaneous leishmaniasis in a sheep from the Eastern Transvaal and human cases of cutaneous leishmaniasis and CanL were reported from the country (Grové 1970; Van der Lugt, Carlyon & Waal 1992; Van der Lugt & Stewart 2004). There has been an increase in the number of imported dogs into the country from countries where CanL is endemic or has reported its occurrence. The main objective of this study was to evaluate the likelihood that CanL could enter and be established in South Africa through importation of live dogs.

## Materials and methods

### *Leishmania* tests

The *Leishmania* test is an IFAT using *L. infantum* antigens (Ag) Antigen slides are prepared from culture suspension of promastigote of *L. infantum* (ITMAP 263 – clone 10) according to the protocol adopted and modified from the OIE Manual (OIE 2016), which mentions the sensitivity as 96% and specificity as 98%. A titre of ≥ 1:50 is considered positive for South Africa. Molecular tests and DNA (deoxyribonucleic acid) sequence analysis were performed as confirmatory tests. A real-time polymerase chain reaction (PCR) capable of detecting and differentiating the *L. donovani* complex (*L. donovani, L. infantum* and *L. chagasi*), the *L. brasiliensis* complex as well as other *Leishmania* spp. (such as *L. major, L. mexicana* and *L. tropica*) was used for diagnostic screening (Schulz et al. 2003). This test is based on the 18S rRNA gene and amplicons were sequenced directly from both directions to obtain consensus sequences. As a positive control, we used the *L. infantum* IFAT Ag. Amplification of the 18S gene was performed using primers 609F and 706R previously published (Maia da Silva et al. 2004) that yield a fragment of ~900 bp (base pair) spanning the V7–V8 hypervariable region. Sequencing was performed at Inqaba Biotech (South Africa), and sequences were analysed using BLASTN analysis (Altschul et al. 1990) and assigned to a species complex based on 100% identity.

### Phlebotomine sandfly species

One ad hoc collection of sandflies from Kuleni, KwaZulu-Natal, was made. Flies were collected using light traps and samples were preserved in 70% alcohol until identification. Sandflies were processed and identified morphologically and genetically as described by Krüger 2017.

### Assessment of risk factors

Our import risk assessment regarding the spread of the disease follows the World Organization for Animal Health formal method of quantitative risk assessment (OIE 2016). The risk assessment is a formal method to deal with hazards and associated risks and has been documented as a step-by-step process. The hazard identification (infectious pathogens, i.e. *Leishmania* parasites) is the first step and considered separately from the risk assessment. The risk assessment process follows three steps: (1) entry (release) assessment that describes the pathways necessary for the introduction of the hazard, (2) exposure assessment (description of pathways necessary for the hazard to occur following entry) and (3) consequence assessment (identification of the consequences of disease entry and establishment such as the effect on human health and animal health).

### Hazard identification

Canine leishmaniasis is an exotic and controlled disease in South Africa. The main purpose of our risk assessment study was to evaluate the likelihood that this exotic CanL could be established in South Africa through importation of live infected dogs.

### Risk factors identified in release assessment (likelihood of entry)

Risk factors identified in release assessment included confirmed diagnosis at port of entry, missed diagnosis at port of entry, imported parasitic infected or uninfected vector ticks, diseases reported in exporting countries, report of the disease in neighbouring and regional countries and infected dogs of unknown origin or illegal entry into the country.

### Risk factors identified in exposure assessment (likelihood of target population to be exposed)

Risk factors identified in exposure assessment included presence of sandfly vectors and other means of transmission such as mechanical or direct transmission (vertical transmission, venereal and dog biting wounds).

### Consequences assessment (likelihood of occurrence and magnitude)

The consequences assessment included disease transmission to canine/wildlife animals and human health (zoonosis).

### Risk assessment

Risk assessment, risk management and risk communication are components of risk analysis. The OIE provides an import risk analysis standard (OIE 2016). The four components of the risk analysis involve hazards identification, risk assessment, risk management and risk communication. In the present study, we have identified the hazards and assessed risks step-by-step but have not covered the risk management and risk communication as adopted by the OIE (2016). However, some aspects of risk management were highlighted to provide guidelines to reduce the risk of introduction of CanL in the country and assist in planning future management policies based on scientific data, published evidence and expert opinion.

A qualitative risk assessment was conducted, and so the likelihood of release and exposure, as well as the magnitude of the consequences, was expressed as negligible, low, moderate and high. A scenario tree was used, detailing the release (entry), exposure and consequence pathways of *Leishmania* introduction into South Africa. The likelihood is a product of all the steps in the scenario tree (Peeler, Reese & Thrush 2015). This assessment is summarised using a risk matrix, which combines the likelihood of the hazard and the consequences of that hazard to produce an overall risk estimate (Desvaux et al. 2016; Dufour et al. 2011). The final risk estimate considers the degree of uncertainty in the available data and scientific evidence (Costard 2008).

### Ethical considerations

The Agricultural Research Council-Onderstepoort Veterinary Research Parasitology Diagnostics Laboratory is accredited by South African National Accreditation System and approved by the Department of Agriculture, Forestry and Fisheries.

## Results & discussion

### Risk factors identified in release assessment (likelihood of entry)

#### Probability of a screening test (immunofluorescence antibody test) missing an infected animal at a port of entry

**FIGURE 1:**
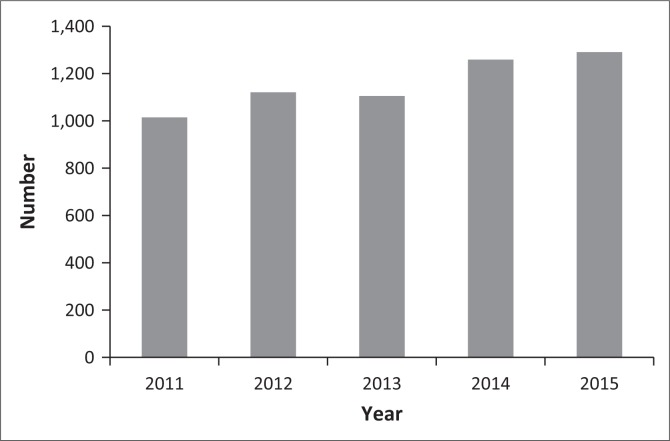
Number of diagnostic IFAT for canine *Leishmania* per year (2011–2015); dogs intended for importation into South Africa.

**FIGURE 2:**
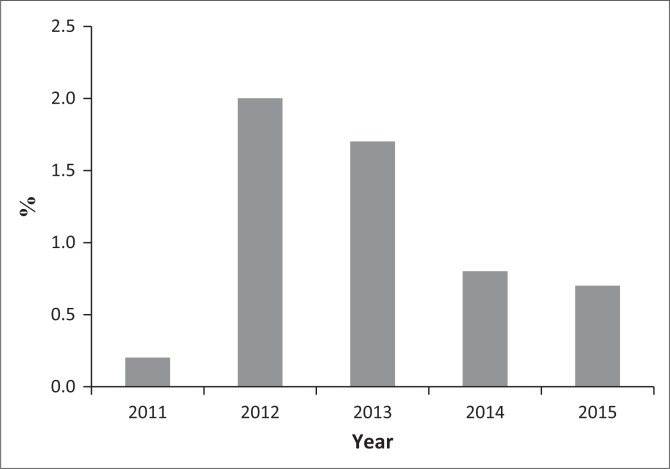
Percentage of *Leishmania* positive IFAT from 2011 – 2015, dogs intended for importation into South Africa.

The number of dogs imported into South Africa during 2011–2015 and tested for the controlled leishmaniasisis is shown in Figure 1. The average number of tests performed per year was 1158. Table 1 shows the number of diagnostic tests performed for the disease (1574 tests) on dogs imported from 44 countries into South Africa. *Leishmania* is reported to be endemic or to occur in 21/44 (47.7%) exporting countries (Table 1). A high percentage of dogs (71.1%) were imported from CanL endemic countries or where the disease has been reported. The yearly percentage of seropositive ranged from 0.2% to 2.0% (Figure 2).

**TABLE 1:** Canine *Leishmania* serological tests (IFAT) performed for dogs for importation into South Africa (1574).

Region	Tests		Exporting countries with disease reported (Ref. shown)	Countries reporting disease who export dogs to South Africa	
	Number	%		Proportion	%
Africa	774	49.0	Zambia^1,2,3,4^, Kenya^3,4,5^, Angola^3,4,6,7^, Namibia^3,4,8,9^, Malawi^3,10^, Nigeria^3,4^, Cameron^3,4^, Democratic Republic of Congo^3,4^	8/15	53.3
Middle East	342	22.6	Qatar^11^, Israel^3^, Kuwait^3^, Oman^3^, Saudi Arabia^3,12^	5/8	62.5
Asia	196	12.3	India^13,14^, China^15^, Turkey^16^	3/12	25.0
Europe	187	11.7	Greece^17^	1/4	25.0
South America	50	3.0	Brazil^18,19,20^, Argentina^3^	2/2	100.0
North America	25	1.5	United States^21^, Canada^21^	2/3	66.7

Ref., reference.

*Source*: Compiled from (1) Campbell et al., 1976; (2) Naik et al., 1976; (3) WHO, 2010; (4) Alvar et al., 2012; (5) Beach et al., 1984; (6) Jimenez et al., 1994; (7) Vilhena et al., 2014; (8) Grové, 1970; (9) Noden & Soni, 2015; (10) Pharoah et al., 1993; (11) WHO, 2015; (12) Salam et al., 2014; (13) Megat et al., 2010; (14) Sharma et al., 2005; (15) Zhi-Biao, 1989; (16) Ok et al., 2002; (17) Frank et al., 1993; (18) Jennings et al., 2014; (19) Harhay et al., 2011; (20) Cardoso et al., 2015; (21**)** Duprey et al., 2006.

#### Missed diagnoses at port of entry

Serology employing the IFAT, which uses whole promastigotes antigen, is highly specific and sensitive for the detection of CanL and is the recommended diagnostic method (OIE 2016). In CanL, high Ab titres are associated with high parasitaemia and disease. Some of the dogs remain seronegative for extended periods of time after infection. This long incubation period results in false negative results, which has an implication in the diagnosis of the disease in imported asymptomatic dogs. Thus, the test may lack sensitivity (about 4%) to detect clinically healthy but infected dogs (Mettler et al. 2005; Oliva et al. 2006).

#### A missed diagnosed case in Durban North, Ethekwini (2012)

A 4-year-old Golden Spaniel male dog was imported into South Africa from Italy in October 2010. It was released from quarantine and moved to Durban. In March 2011 (6 months later), the dog was tested for *Leishmania* antibodies using the IFAT and the results were positive at a 1/50 dilution. A re-test was carried out in June 2012 (15 months after the first test) and the result was positive at 1/800 dilution. The dog was euthanised on 30 August 2012 (22 months after importation) and the post-mortem confirmed infection with *Leishmania* (Anon 2012).

**A missed diagnosed case in Cape Town (2014):** Two dogs, a 4-year-old male and a 6-year-old female Bull Terrier, were imported from Angola into South Africa on 5th of February 2014 and landed at Cape Town. Both tested IFAT negative for *Leishmania* 76 days before importation and were not tested again within the prescribed 30-day period before movement. The dogs remained negative on testing at destination and were released. The female dog was presented 6 days later at the veterinary clinic with a generalised nodular skin condition. The biopsy samples and Giemsa’s stained smears showed *Leishmania* amastigotes intracellularly in macrophages and cutaneous fibroblasts which confirmed the diagnosis (Grewar & Brady 2014).

### Autochthonous leishmaniasis cases

#### *Leishmania* case study in Durban (2015)

A3-year-old Rottweiler male dog, with an uncertain history, was presented at a private veterinary clinic in Durban in February 2015. The patient showed general poor body condition, loss of body weight, scruffy coat, bilateral ocular discharge, hair loss on tips of tail and ears without pruritis being evident at the time. Two months later, the skin showed exfoliative dermatitis, multiple areas of localised alopecia, skin thickening and ulceration particularly on pressure points, pin bones and elbows. Other signs included generalised lymphadenopathy.

Isolation and identification of parasite from lymph node, bone marrow and spleen aspirate were successful in cell culture. The bone marrow cell culture demonstrated extensive *Leishmania* growth, with typical *Leishmania* cells in Giemsa stained smears. A sample of the organism tested positive by PCR. Sequencing of the cell cultures obtained from the dog and the standard cell culture used for IFAT indicated that both were 100% identical to each other and to the *Leishmania donovani*/*chagasi*/*infantum* complex.

#### *Leishmania* case reports in dogs in Durban (1964) and Free State (1987)

Leishmaniasis was diagnosed twice in dogs: in 1964 in a dog from Durban whose life history was uncertain and in 1987 in a dog from the Free State which had never left the country (Van der Lugt & Stewart 2004).

#### Leishmaniasis reported in sheep (1992)

A case of cutaneous leishmaniasis was reported in a sheep from the Eastern Transvaal (Van der Lugt et al. 1992). The skin lesions were described and the amastigote stage of *Leishmania* species was identified.

### Imported parasitic infected/uninfected vector ticks

Tick-associated transmission of *Leishmania* has been reported (Mckenzie 1984) (see risk factors identified in ‘exposure’ assessment). Tick inspection is the responsibility of the veterinary authority at entry ports.

### Leishmaniasis reported in exporting countries

The dogs came from 8/15 African countries with history of the disease, 5/8 countries in the Middle East, 3/12 countries in Asia, 1/4 of European countries, from the two South American countries, Brazil and Argentina, as well as from the United States and Canada (2/3) (Table 1).

### Report of the disease in neighbouring countries or countries in the region

Leishmaniasis has been reported in several countries neighbouring or in the southern region; Angola, Botswana, Malawi, Mozambique, Namibia and Zambia (Alvar et al. 2012; World Health Organization 2010). Autochthonous cutaneous leishmaniasis identified as belonging to the *L. tropica* group has been reported in 34 cases in Namibia since the 1970s. *Leishmania* parasites have also been isolated from hyraxes (*Procavia capensis*) and from naturally infected *Phlebotomus rossi* sandflies, which are possible vectors of the human disease (Campbell, Gordon & Emms 1979; Grové 1970, 1989; Grové & Van Dyk 1974; Noden & Soni 2015; Rutherfoord & Uys 1978). If autochthonous leishmaniasis is not monitored, it can suddenly become an epidemic should ecological and environmental conditions change (Noden & Van der Colf 2013). A nurse was evaluated for cutaneous leishmaniasis, after showing skin lesions 3 weeks after travel to Botswana and visiting a game reserve. The biopsy revealed promastigotes of *L. tropica* (Schwartz et al. 2012). A 26-year-old man from Angola with no history of travel outside the country had visceral leishmaniasis. The parasite was isolated and biochemically characterised using molecular tools and identified as *L. infantum*, a parasite not endemic to this region (Jimenez et al. 1994). Recently, a dog that had never left Angola nor had its parents was found seropositive with DAT titres of 800 and ≥6400 and was also found to be PCR-positive and confirmed to be infected with *L. infantum* by DNA sequence analysis. This case is strongly suggestive of an autochthonous infection (Vilhena et al. 2014). Two autochthonous cases of cutaneous leishmaniasis in Zambia were described in humans, both of whom also had tuberculosis. Amastigotes were cultured from blood and identified in skin, bone marrow, liver and spleen (Naik et al. 1976). Two cases of cutaneous leishmaniasis in Malawi were diagnosed by histopathology in 1 year. The authors suggested that the infection may be more prevalent in this region than what was previously thought (Pharoah et al. 1993).

### Risk factors identified in exposure assessment (likelihood of the target population to be exposed)

#### Presence of sandfly vectors (biological transmission)

Female Phlebotomine sandflies are the only vectors of *Leishmania* spp. and also the main mode of parasite transmission (Dantas-Torres et al. 2012; Killick-Kendrick 1990).

#### Sandfly situation in South Africa

**Identification of sandflies in Kuleni, KwaZulu-Natal:** Out of 13 sandflies (11 females and 2 males), 10 were dissected for micromorphological identification. Of these, nine were *Grassomyia* (formerly *Sergentomyia*) *squamipleuris*, one male could only be specified as *Sergentomyia* spec., but not *G. squamipleuris. G. squamipleuris* is supposedly a reptile-feeder and not known as a mammalian *Leishmania* vector. It has been reported before from Transvaal and Zululand (De Meillon 1955). On the other hand, very little is known about the host preferences for most sandfly species, but it was suggested that *Sergentomyia schwetzi* may bite dogs (Senghor et al. 2016).

**Records of *Phlebotomus* sp.:** Research into the sandfly populations of South Africa and of the Southern African neighbouring countries has never been carried out systematically and species records came only from ad hoc and limited surveys. The last known record of Phlebotomines in South Africa dates back to 1987 (Braack et al. 1981; Davidson 1980, 1981, 1983, 1987; Lewis 1967, 1971; Zielke 1971). The first phlebotomine sandfly collection from Botswana, southern Africa, was carried out in 2014/2015 (Krüger 2015). During a pilot survey in the north of the country, 41 specimens were collected, of which 37 were morphologically and genetically identified to be species of the genera *Sergentomyia* and *Phlebotomus* (Krüger 2017). The sandfly fauna of southern Africa accounts for about 49 species (Krüger 2015) and 17 different species are known so far from South Africa and of South West Africa/Namibia (Zielke 1971). Only a few specimens of the genus *Phlebotomus* were caught whereas the majority belongs to the genus *Sergentomyia*.

**Vectorial capacity of phlebotomine sandflies:** *Phlebotomus rodhaini,* a putative vector of zoonotic leishmaniasis, was recorded recently from Botswana (Krüger 2017) and this species has also been reported to occur in Namibia and South Africa (Davidson 1981). *Phlebotomus rossi* was reported to be infected with *Leishmania* parasites in Namibian loci of cutaneous leishmaniasis and its distribution covers Namibia, South Africa and Zimbabwe (Davidson 1987; Killick-Kendrick 1990). The status of *P. rossi*, the suspected vector of cutaneous leishmaniasis in South West Africa, was revised by Lewis and Legder (1976).

Most *Sergentomyia* spp. feed preferentially on cold-blooded vertebrates, being proven vectors of reptile *Leishmania* species. Their possible role in the circulation of mammalian leishmaniasis in the Old World has been considered where *Leishmania* DNA and the parasites have been identified in several species, while some species were reported to feed on mammals, including man (Ayari et al. 2016; Berdjane-Brouk et al. 2012; Maia et al. 2015; Senghor et al. 2016). Recently, three species of the genus *Sergentomyia* were demonstrated to have a high rate of *L. infantum*-positive females under natural conditions. This finding indicated that these species were the natural vectors of CanL in the Mont-Rolland area of Senegal and contradicts the notion that species of the *Phlebotomus* genus in the Old World are the only vectors of leishmaniasis (Senghor et al. 2016). Three records of sandflies biting a man in South West Africa show that further observations on these nocturnal insects in South Africa are necessary (Lewis 1971). However, several aspects of the fly–parasite relationships must be elucidated to confirm the fly species as a vector of leishmaniasis, such as obtaining data on the species richness, abundance, biting and infection rates, taking of blood meals from reservoir hosts (e.g. rodents) as well as from humans to confirm using xenodiagnostic attempts (Maia & Depaquit 2016; Pech-May et al. 2010).

### Ticks, mechanical, and direct mode of transmissions

#### Tick transmission

Sandflies are the accepted biological vectors of *Leishmania* parasites. Currently, other modes of disease transmission have been reported in the literature. Reports of ticks as vectors of *Leishmania* were studied by scientists in different disease-endemic areas. *Rhipicephalus sanguineus* has long been suspected to transmit *Leishmania infantum* in studies carried out in laboratory and natural conditions. The role of the brown dog ticks, *R. sanguineus*, as vectors of *Leishmania* is highlighted herein.

Conclusive evidence of xenodiagnosis was reported by McKenzie (1984). In that study, a tick pick-up and transmission using *R. sanguineus* nymphs fed on two naturally infected dogs and the subsequent adult stage were fed on two uninfected dogs. Transmission attempt was performed on one recipient infected dog concluded that *R. sanguineus* was able to transmit *L. infantum* to a susceptible dog through normal feeding. Infections were monitored in dogs using serology, xenodiagnosis with ticks and direct culture of bone marrow and lymph node aspirates. Based on this study, Dantas-Torres (2011) concluded that *R. sanguineus* was able to transmit *L. infantum* to a susceptible dog through normal feeding, and as such demonstrated that the tick-vector theory deserves attention.

The study by Solano-Gallego et al. (2012) has shown high prevalence of *L. infantum* DNA in *R. sanguineus* (males and females) removed from *L. infantum* seropositive and seronegative dogs. This study was also supported by the results obtained by Colombo et al. (2011). These authors showed that live parasites were detected in newly moulted *R. sanguineus* adult ticks obtained as nymphs, which engorged on dogs in the endemic area, and thus could confirm transstadial transmission. Dantas-Torres et al. (2010a) reported for the first time the retrieval of *L. infantum* kDNA in salivary glands of *R. sanguineus* ticks and recommended further studies to assess the competence of ticks as vectors of *Leishmania* parasites. This was followed by studies of Medeiros-Silva et al. (2015) who reported the presence of the parasite DNA in the intestines and salivary glands of *R. sanguineus* and viable *L. infantum* could be successfully isolated. In an experimental injection of *L. infantum* promastigotes into the haemocoel through the coxa of engorged females, the subsequent eggs and larvae were found positive for *L. infantum* kDNA (Dantas-Torres et al. 2010b; Dantas-Torres, Latrofa & Otranto 2011). These authors concluded that their results have shown, for the first time, the transovarial passage of *L. infantum* kDNA in *R. sanguineus*. Moreover, the potential transovarial and transstadial passage of kDNA through ticks was confirmed by PCR (Dabaghmanesha et al. 2016). *R. sanguineus* removed from *Leishmania*-infected dogs was found to be infective to hamsters (Coutinho et al. 2005). All these results highlight the potential of *R. sanguineus* as a vector of *L. infantum*. However, in his review article entitled ‘Ticks as vectors of *Leishmania* parasites’, Dantas-Torres (2011) emphasised the need for further research to better understand the participation of *R. sanguineus* in the epidemiology of leishmaniasis.

**Presence of the brown dog tick *R. sanguineus* in the country:** In South Africa, all life stages of *R. sanguineus* feed on domestic dogs (Walker, Keirans & Horak 2000). The tick was collected from dogs in the Northern Cape, Eastern Cape, North West province and the High Veld (Bryson et al. 2000; Horak 1995; Matthee et al. 2010; Nyangiwe, Horak & Bryson 2006). It is a known vector of *Ehrlichia canis* and *Babesia vogeli* in South Africa.

#### Other modes of transmission

Other modes of transmission included transplacental transmission (Boggiatto et al. 2011; Rosypal et al. 2005). Boggiatto et al. (2011) described the first and novel report of disseminated *L. infantum* parasites as identified by qPCR in 8-day-old pups born to a naturally infected seropositive dog with no travel history. This is considered the first report of vertical transmission of *L. infantum* in naturally infected dogs in North America, emphasising that this novel means of transmission could possibly sustain infection within populations. Other modes of direct transmission were through dog bite wounds (Duprey et al. 2006; Naucke, Amelung & Lorentz 2016), venereal transmission (Silva et al. 2009), blood transfusions (Freitas et al. 2006; Owens et al. 2001) and haematophagous insects and fleas (Colombo et al. 2011; Coutinho & Linardi 2007; Dantas-Torres 2006; Daval et al. 2016; De Morais et al. 2013; Ferreira, Fattori & Lima 2009; Gustavo et al. 2010; Otranto & Dantas-Torres 2009; Seblova et al. 2014; Slama et al. 2014). The authors concluded that the occurrence and persistence of limited CanL foci (e.g. in households or in kennels) is a threat for further spread of the disease in non-endemic areas should competent vectors be introduced.

### Consequences assessment (likelihood of occurrence and magnitude)

#### Disease transmission to dogs/wildlife animals

*Leishmania* parasites are zoonotic multi-host parasites, which may be maintained in several mammalian species in nature. Roque and Jansen (2014) reviewed the mammalian species known to be infected with *Leishmania* spp. in the Americas, highlighting those that can maintain and act as a source of the parasite in nature. These host reservoirs were presented separately in each of seven mammal orders; Marsupialia, Cingulata, Pilosa, Rodentia, Primata, Carnivora and Chiroptera responsible for maintaining *Leishmania* species in the wild (more than 80 species of animals).

Ashford et al. (1973) proved that the hyraxes *Procavia habessinica* and *Heterohyrax brucei* are the natural reservoirs of cutaneous leishmaniasis in highland Ethiopia. Further cases of cutaneous leishmaniasis were diagnosed in Namibia by finding amastigotes in sections of excised lesions from hyraxes. A culture of tissue from the tip of the nose of one animal in diphasic blood-agar medium showed active promastigotes 14 days after inoculation. Iberian hares (*Lepus granatensis***)** were recently deemed responsible for an outbreak of human leishmaniasis in Spain (Carillo, Moreno & Cruz 2013; Ruiz-Fons, Ferroglio & Gortázar 2013). Jackals and foxes may play a role in the spread of zoonotic *L. tropica* (Talmi-Frank et al. 2010). Faiman et al. (2013) reported that two species of rodents, the Levant voles (*Microtus guentheri*) and Tristram’s jirds (*M. tristrami*) as reservoirs of *L. major* in Israel.

### Human health (zoonosis) in South Africa and neighbouring countries

The classification of visceral and cutaneous forms of leishmaniasis as observed in human disease cannot be applied to the infection in other mammals. Dogs infected with *L. infantum* present viscero-dermal disease, where parasite isolation is common even from intact skin. Moreover, *Leishmania* species associated with human cutaneous infection have been observed in rodent viscera (Roque et al. 2010).

Four cases of cutaneous leishmaniasis were reported from the Republic of South Africa and from South West Africa/Namibia in 1970 (Grové 1970). In this report, it was stated that two of patients had never been out of South Africa or South West Africa; and the other two probably contracted the infection in South or South West Africa. The lesions were typical nodules or ulcers and the diagnosis was proven by histology which also showed typical intracellular amastigotes of *Leishmania.* This was the first report of cutaneous leishmaniasis in southern Africa. Again, cutaneous leishmaniasis from South West Africa/Namibia was reported in 1978 and the authors made the following comment ‘This adds a further dimension to the characterisation of this disease in southern Africa’ (Grové 1989; Rutherfoord & Uys 1978). In the years 1974 and 1976, autochthonous cases of human cutaneous leishmaniasis were reported in Zambia (Campbell, Gordon & Emms 1976; Grové & Van Dyk 1974; Naik et al. 1976). Two cases of cutaneous leishmaniasis in Malawi were diagnosed by histopathology in northern Malawi in 1 year (Pharoah et al. 1993). These authors also made the following comment ‘the *Leishmania* species responsible could not be identified, but the infection may be more prevalent in this region than previously thought’. A 26-year-old man from Angola with no history of travel outside the country was diagnosed with typical symptoms of visceral leishmaniasis. The parasite was isolated and characterised using both kinetoplast DNA and nuclear DNA probes and showed a strong homology with *L. infantum* (Jimenez et al. 1994). A patient contracted cutaneous leishmaniasis 3 weeks after travel to Botswana and a visit to a game reserve (Schwartz et al. 2012).

### Risk assessment

Risk assessment, risk management and risk communication are components of risk analysis. A qualitative risk assessment was conducted, and so the likelihood of release and exposure as well as the magnitude of the consequences were expressed in terms of negligible, low, moderate and high. A scenario tree was used, detailing the release (entry), exposure and consequence pathways of *Leishmania* introduction into South Africa. The likelihood is a product of all the steps in the scenario tree (Peeler et al. 2015). This assessment is summarised using a risk matrix, which combines the likelihood of the hazard and the consequences of that hazard to produce an overall risk estimate (Desvaux et al. 2016; Dufour et al. 2011). The final risk estimate considers the degree of uncertainty in the available data and scientific evidence (Costard 2008).

**FIGURE 3:**
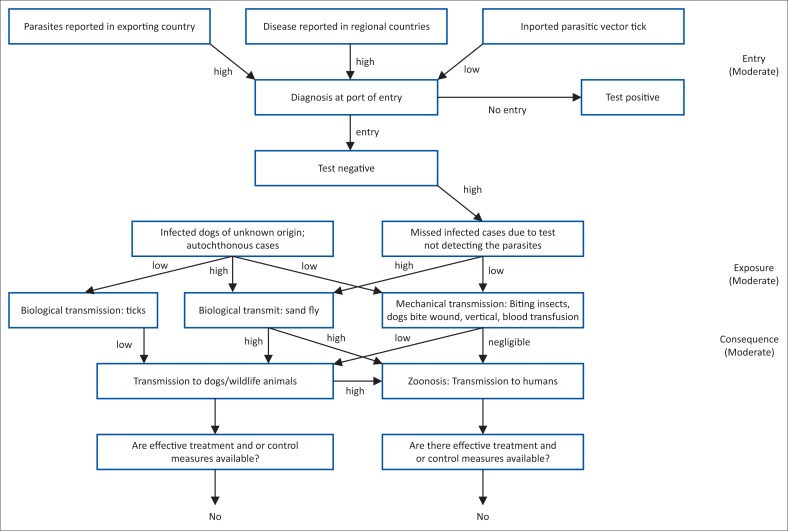
Risk pathway and probability (negligible to high) for entry (release), exposure and consequence assessment for introduction of canine leishmaniasis.

Figure 3 depicts the ‘risk pathway’ scenario tree for the introduction of *Leishmania*. For entry, CanL is reported in exporting countries, in neighbouring and regional countries and as such, the risk is considered high. After diagnosis, if the test result is negative, there is a probability that this is because of a missed case and not necessarily because the test is true negative. If the sandfly vector is present (two putative vector species present), then this leads to a high chance of transmission to dogs, wildlife and zoonotic cases arising. If transmission was to occur through mechanical means (venereal, vertical or through blood transfusion) then, transmission to dogs and other wildlife would be low and confined in a dog population however, negligible in humans. If autochthonous and cases of infected dogs, assumed to be of unknown origins or illegal importation, transmission by sandflies and therefore to dogs/wildlife and humans would be high. In a transmission scenario is to occur in dogs, wildlife and humans, there are no effective control or treatment measures currently in place to effectively maintain the spread. Considering these different risk factors, a moderate likelihood of the introduction of *Leishmania* is expected. This is further summarised in Table 2, which shows the likelihood of the *Leishmania* entry and the consequence of this hazard, which in our case are both moderate and this results in a moderate risk estimate. For a risk assessment to be complete, uncertainty about the available data and scientific evidence should be given. Table 3 shows the degrees of uncertainty in available sources. Reliable and complete data needed for the quantification of the risk of introduction of *Leishmania* is lacking but there is strong scientific evidence available in multiple references, although some conclusions reported by review authors vary. A moderate degree of uncertainty is concluded from our risk assessment and therefore the final risk estimate is given as moderate.

**TABLE 2:** Combination risk matrix of the likelihood of exposure and entry of *Leishmania* and the consequence assessment.

Consequence	Likelihood			
	Negligible	Low	Moderate†	High
Negligible	Negligible	Low	Low	Moderate
Low	Low	Low	Moderate	Moderate
Moderate†	Low	Moderate	Moderate†	High
High	Moderate	Moderate	High	High

† , The present study.

*Source*: Adapted from Dufour, B., Plée, L., Moutou, F., Boisseleau, D., Chartier, C., Durand, B. et al., 2011, ‘A qualitative risk assessment methodology for scientific expert panels’, *Scientific and Technical Review of the Office International des Epizooties (Paris)* 30, 673–681. https://doi.org/10.20506/rst.30.3.2063 and Desvaux, S., Nguyen, C.O., Vu, D.T., Henriquez, C., Ky, V.D., Roger, F. et al., 2016, ‘Risk of introduction in Northern Vietnam of HPAI Viruses from China: Description, patterns and drivers of illegal poultry trade’, *Transboundary and Emerging Diseases* 63, 389–397. https://doi.org/10.1111/tbed.12279.

**TABLE 3:** Degree of uncertainty in the quality and availability of data and scientific evidence in relation to qualitative risk assessment.

Uncertainty Category	Interpretation
Low	Complete data available; strong evidence provided in multiple references and authors report similar conclusions
Moderate†	Incomplete data available; lack of surveillance data; strong evidence provided in multiple references; conclusions reported by review authors vary
High	Little or no data available; evidence provided in unpublished reports or observations, authors give conclusions that vary considerably

† , The present study.

*Source*: Adapted from Costard, S., 2008, *Introduction to risk analysis and risk assessment*, Royal Veterinary College, University of London, London, viewed 04 December 2018, from https://assets.publishing.service.gov.uk/media/57a08bd1ed915d3cfd000f68/WKS081002_Annex5.pdf.

## Conclusion and recommendations

Not much attention has been given to the reported and published autochthonous leishmaniasis cases involving rural dogs and humans since 1970 in South Africa and southern African countries in general. It seems as if the risk has either not been considered or has been underestimated or neglected. Autochthonous leishmaniasis ecology and epidemiology should be studied under current conditions of climatic and environmental change.

Considering that only few reports of leishmaniasis infection in wildlife host reservoirs are available in South Africa, long-term investigations must be undertaken in this field. Attention should be given to surveillance of the reservoir infection in rodents and hyraxes in view of a higher incidence of infection in these animal species encountered in Africa.

The main route of transmission of CanL parasite to mammals is via the bite of the female phlebotomine sandfly (Killick-Kendrick 1990). Two putative and suspected vector species have been reported to occur in South Africa, Namibia and Botswana: *P. rodhaini* and *P. rossi* (Davidson 1981, 1987; Killick-Kendrick 1990; Krüger 2017; Lewis & Legder 1976). The abundance and vector capacity of the two species have not been demonstrated. The occurrence and persistence of limited CanL foci (direct transmission, vertical, venereal and blood transfusion) is a threat for further spread of the disease in non-endemic areas should the competence of these putative vectors be established. Where sandfly populations are likely to have a lower vectorial capacity than in endemic areas because of lower vector densities, the probability of establishment following introduction of an infected dog remains high, according to the model (EFSA Panel Animal Health and Welfare 2015). Only few authors in South Africa and southern Africa have made contributions to the studies on sandflies. This resulted in very few collections being made, and currently there is not much information about the distribution of phlebotomine sandflies and their ecology, biology and disease relationships. This emphasises the need for improving our knowledge of the vector competence of these two sandfly species and of the distribution and abundance of other known vectors present in Africa.
